# Decoding isonicotinylation-associated patterns in neutrophil chronic obstructive pulmonary disease: evidence from integrative bioinformatic-driven multi-omics and *in vitro* validation

**DOI:** 10.3389/fphys.2026.1870580

**Published:** 2026-06-09

**Authors:** Zizhong Wang, Yuan Li, Mengqi Zhou, Min Xiang, Jiangtao Lin

**Affiliations:** 1 Graduate School, Beijing University of Chinese Medicine, Beijing, China; 2 Department of Pulmonary and Critical Care Medicine, China-Japan Friendship Hospital, Beijing, China

**Keywords:** artificial intelligence, chronic obstructive pulmonary disease, isonicotinylation, multi-omics, neutrophil

## Abstract

**Background:**

Chronic obstructive pulmonary disease (COPD) is driven by complex inflammatory processes in which neutrophil dysregulation plays a central role. Isonicotinylation (Kinic), a novel lysine acylation modification linked to cellular metabolism, has not yet been clearly associated with COPD.

**Methods:**

We employed an artificial intelligence (AI)-driven, multi-omics framework. First, the Limma, WGCNA, and CIBERSORT algorithms were used to identify a Kinic- and neutrophil (KN)-associated shared molecular signature in the peripheral blood bulk profiles of COPD patients. Next, NMF and explainable machine learning identified molecular subgroups and developed a diagnostic model for COPD patients based on the KN-associated gene signature. In addition, a KN-associated pathogenic hub factor was identified, and its molecular and immune signatures in COPD were assessed using a cutting-edge analytical framework in spatial and temporal manners. An AI-based drug screening platform (DrugReflector) and molecular docking were used to identify therapeutic candidates. Finally, Lymphotoxin-beta (LTB) expression was validated *in vitro* using q-RT-PCR assays.

**Results:**

We identified a robust five-gene KN-associated signature that demonstrated diagnostic and patient-stratification potential in COPD. LTB was identified as an upregulated Kinic-associated hub gene mainly distributed in neutrophils and involved in the regulation of COPD pathogenesis. Notably, BRD-K97481123 was identified as a potential LTB-targeting compound for the treatment of COPD.

**Conclusion:**

This study unveils a novel KN-associated molecular axis in COPD pathogenesis, with LTB as a neutrophil-centric pathogenic factor. This axis provides a framework for patient stratification, offers a promising diagnostic biomarker, and identifies a potential therapeutic target, thereby linking a novel metabolic modification to neutrophilic inflammation in COPD.

## 1 Introduction

Chronic obstructive pulmonary disease (COPD) is a prevalent, progressive respiratory disorder characterized by persistent airflow limitation and chronic inflammation, leading to significant morbidity and mortality worldwide (Ritchie and Wedzicha, 2020). COPD pathogenesis involves a complex interplay of environmental exposures, aberrant immune responses, and tissue remodeling (Hattab et al., 2016). Beyond the classical involvement of neutrophils, macrophages, and lymphocytes, emerging evidence highlights the role of post-translational modifications (PTMs) in modulating protein function and disease progression (Qian et al., 2023).

Isonicotinylation (Kinic) is a recently characterized lysine acylation in which an isonicotinoyl group is transferred onto lysine residues, potentially altering protein stability, interactions, and activity (Jiang et al., 2021). More specifically, for histone proteins, the acetyltransferases CREB-binding protein (CBP) and P300 can mediate Kinic modification of histones, thereby relaxing chromatin structure and promoting the transcription of genes, such as those that regulate the PI3K-AKT-mTOR in liver cancer (Jiang et al., 2021). In non-histone proteins, CBP and Tip60 can mediate Kinic modification of the SMAD3 protein, subsequently activating the TGFβ pathway (Li et al., 2024). In addition, recent studies have suggested that Kinic may serve as an efficient predictive and therapeutic marker for liver cancer patients (Zhou et al., 2026). This modification, which is linked to cellular metabolic states, has been implicated in cancer but remains uninvestigated in COPD (Li et al., 2024). Concurrently, neutrophils are widely recognized as the predominant inflammatory effector cells in the COPD airway, and their dysregulation is a hallmark of disease pathogenesis (Chen et al., 2024). In the lungs of patients with COPD, persistent exposure to cigarette smoke and other noxious particles drives a chronic inflammatory milieu that recruits neutrophils from the circulation into the airways and parenchyma (Kapellos et al., 2023). Once activated, these infiltrating neutrophils exert their pathogenic effects through multiple mechanisms, including the release of serine proteases, matrix metalloproteinases, and reactive oxygen species, which collectively degrade the extracellular matrix, damage the alveolar epithelium, and impair lung repair mechanisms (Yan et al., 2022).

To address this gap, we conducted an integrative multi-omics study leveraging artificial intelligence (AI) to delineate the coordinated role of Kinic-neutrophil biology in COPD. We aimed to identify a shared isonicotinylation-neutrophil (KN)-associated molecular signature in COPD patients, along with its corresponding diagnostic, patient-stratifying, and therapeutic potential.

## 2 Materials and methods

### 2.1 Source of data

Bulk RNA-sequencing datasets from the peripheral blood of COPD patients and healthy controls (HC) were retrieved from the Gene Expression Omnibus (GEO) database. GSE42057 served as the internal set 1. GSE44962 served as the internal set 2. GSE77344 was designated as the training set, and GSE148871 served as the independent validation set. GSE42057 comprises 94 COPD samples and 42 HC samples. GSE55962 comprises 82 COPD samples and 24 HC samples. GSE77344 comprises 172 COPD samples and 20 HC samples. GSE148871 comprises 152 COPD samples and 32 HC samples. Batch effects were assessed and corrected using the sva and Limma R packages (Ritchie et al., 2015). In addition, the Kinic-associated signature was obtained from the relevant literature.

### 2.2 Limma analysis

Differential expression analysis between the COPD and HC groups in GSE42057 was performed using the Limma R package (Ritchie et al., 2015). Genes with a |log2 fold change| > 0.5 and a p-value < 0.05 were defined as differentially expressed genes (DEGs). The intersection of the Kinic-related gene set with the DEGs yielded the Kinic-related DEGs. Next, the Kinic-associated DEGs were analyzed using KEGG and GO enrichment analyses with the clusterProfiler R package, with reference to the KEGG and GO gene sets downloaded from the MSIGDB database (Yu et al., 2012).

### 2.3 CIBERSORT and WGCNA analyses

The relative abundance of neutrophils in samples from GSE55962 was estimated using the CIBERSORT algorithm in R (Chen et al., 2018). WGCNA was performed on the same dataset using the WGCNA R package to construct a scale-free co-expression network (Langfelder and Horvath, 2008). A soft-thresholding power was selected to achieve a scale-free topology (Langfelder and Horvath, 2008). Modules were identified via hierarchical clustering, and the module whose eigengene showed the highest absolute correlation with the neutrophil infiltration score was selected as the neutrophil-correlated module (Langfelder and Horvath, 2008). Genes from this module were extracted and then intersected with Kinic-associated DEGs to obtain KN-associated DEGs. 

### 2.4 Systemic machine learning model construction

A comprehensive 132-model machine learning workflow was implemented using the caret and glmnet packages in R on the training set (GSE77344) and independent validation set (GSE148871) (Wu et al., 2024). The 132-model machine learning framework typically represents a comprehensive grid-search approach that systematically combines multiple classification algorithms with various feature-selection methods and preprocessing strategies. Combinations of these algorithms were applied to the MetaGSE for signature selection and model construction using 10-fold cross-validation (Wu et al., 2024). The optimal model was selected based on the highest mean area under the curve (AUC) index (Wu et al., 2024). Its performance was rigorously evaluated on the independent validation set (GSE148871) using ROC and PR curves via the pROC package in R (Robin et al., 2011). A nomogram coupled with calibration was constructed for clinical translation using the rms package in R (Lin et al., 2024). SHAP analysis was applied to interpret the model and identify the feature (gene) with the greatest contribution, which was designated as the hub gene (Zhou et al., 2026).

### 2.5 NMF analysis

To uncover molecular heterogeneity among COPD patients, we performed non-negative matrix factorization (NMF) based on the expression profiles of the KN-associated signature genes in COPD samples from the training set (GSE77344) using the NMF R package (Cai et al., 2023). NMF decomposes the non-negative gene expression matrix into two lower-dimensional, non-negative matrices, revealing latent molecular patterns that define patient subgroups (Cai et al., 2023). The resulting coefficient matrix from the NMF decomposition was used to assign each COPD sample to either Subtype 1 (C1) or Subtype 2 (C2) (Cai et al., 2023). Subsequently, these identified subtypes were compared for differences in the expression levels of the KN-associated gene signature using the ggplot2 package in R (Gustavsson et al., 2022). Immune cell infiltration profiles were estimated using the CIBERSORT package in R (Chen et al., 2018). The enrichment of pathways was assessed using GSEA with the clusterProfiler package in R (Yu et al., 2012).

### 2.6 Single-cell analysis

The scRNA-seq data (GSE167295, including three COPD peripheral blood samples) were acquired from the GEO database and processed using the Seurat pipeline in R (Butler et al., 2018). Quality control (QC) filtered out low-quality cells (Butler et al., 2018). Dimensionality reduction was performed using PCA, followed by clustering and visualization with UMAP and t-SNE (Butler et al., 2018). Cell types were annotated using canonical marker genes with the singleR package in R (Cao et al., 2023). The expression distribution of the hub gene was visualized across all cell types. Cell communication patterns among various cell types were assessed using the CellChat package in R (Jin et al., 2021). Next, the metabolic pathway changes among various cell types were assessed using the scMetabolism algorithm in R (Argüello et al., 2020). Pseudotime trajectory analysis of neutrophils was conducted using Monocle2 to infer differentiation states (Fang et al., 2022). A virtual knockout of the hub gene in the neutrophil cluster was simulated using the scTenifoldKnk package in R, and the downstream regulatory network and functional impact of the hub gene were assessed by KEGG and GO enrichment analyses using the clusterProfiler R package, with reference to the KEGG and GO gene sets downloaded from the MSIGDB database and the GENEMINIA database (Yu et al., 2012; Osorio et al., 2022). AUCELL was performed to identify Kinic patterns among various cell types in accordance with the Kinic-associated gene list (Chen et al., 2025).

### 2.7 Drug screening analysis

The deep learning platform DrugReflector was employed to screen the Connectivity Map (cMAP) database for compounds predicted to reverse the COPD gene expression signature relative to that of HCs derived from GSE77344 (DeMeo et al., 2025). The top candidate compound was selected (DeMeo et al., 2025). Molecular docking simulations between the identified hub protein and the candidate drug were performed using AutoDock Vina software to predict binding affinity and pose, which were visualized with PyMOL software (Liu et al., 2024).

### 2.8 *In vitro* estimation

To experimentally validate the functional role of LTB in neutrophils in the context of COPD inflammation, we designed *in vitro* assays using a neutrophil model system. Given the challenges associated with transfecting primary human neutrophils, we employed the HL-60 cell line (ATCC, USA), a human promyelocytic leukemia cell line that can be differentiated into N1 neutrophil-like cells. HL-60 cells were cultured in an RPMI-1640 medium (Gibco, USA) supplemented with 20% heat-inactivated fetal bovine serum (FBS, Gibco, USA) and 1% penicillin-streptomycin (Gibco, USA). To induce neutrophil differentiation, the cells were treated with 1.25% dimethyl sulfoxide (DMSO, Sigma-Aldrich, USA) for 5–7 days. Differentiation was confirmed by assessing morphological changes. N1-like neutrophil was designated as the COPD group, and the primary neutrophil served as the control group. Total RNA was extracted from HL-60 using TRIzol Reagent (Takara, China). cDNA was then synthesized using a PrimeScript RT Reagent Kit (Vazyme, China). q-RT−PCR was carried out using TB Green Premix Ex Taq II (Vazyme, China) following the manufacturer’s instructions. The primer sequences (5’→3’) employed in this study are provided below.

LTB:

F: 5’-GCCACCACGCTCTTCTC-3’,

R: 5’-TGGTGGCTTTGTTGATGTTC-3’.

GAPDH:

F: 5’-GGAGCGAGATCCCTCCAAAAT-3’.

R: 5’-GGCTGTTGTCATACTTCTCATGG-3’.

### 2.9 Statistical analysis

Statistical analyses were performed using R (version 4.2.2) or GraphPad Prism (version 9.0). For two-group comparisons, a Student’s t-test or a Mann-Whitney U test was applied, as appropriate. For multiple-group comparisons, one-way ANOVA with a Tukey *post-hoc* test was used. A p-value < 0.05 was considered statistically significant.

## 3 Results

### 3.1 Identification of Kinic-related DEGs in COPD patients

**Figure 1:**
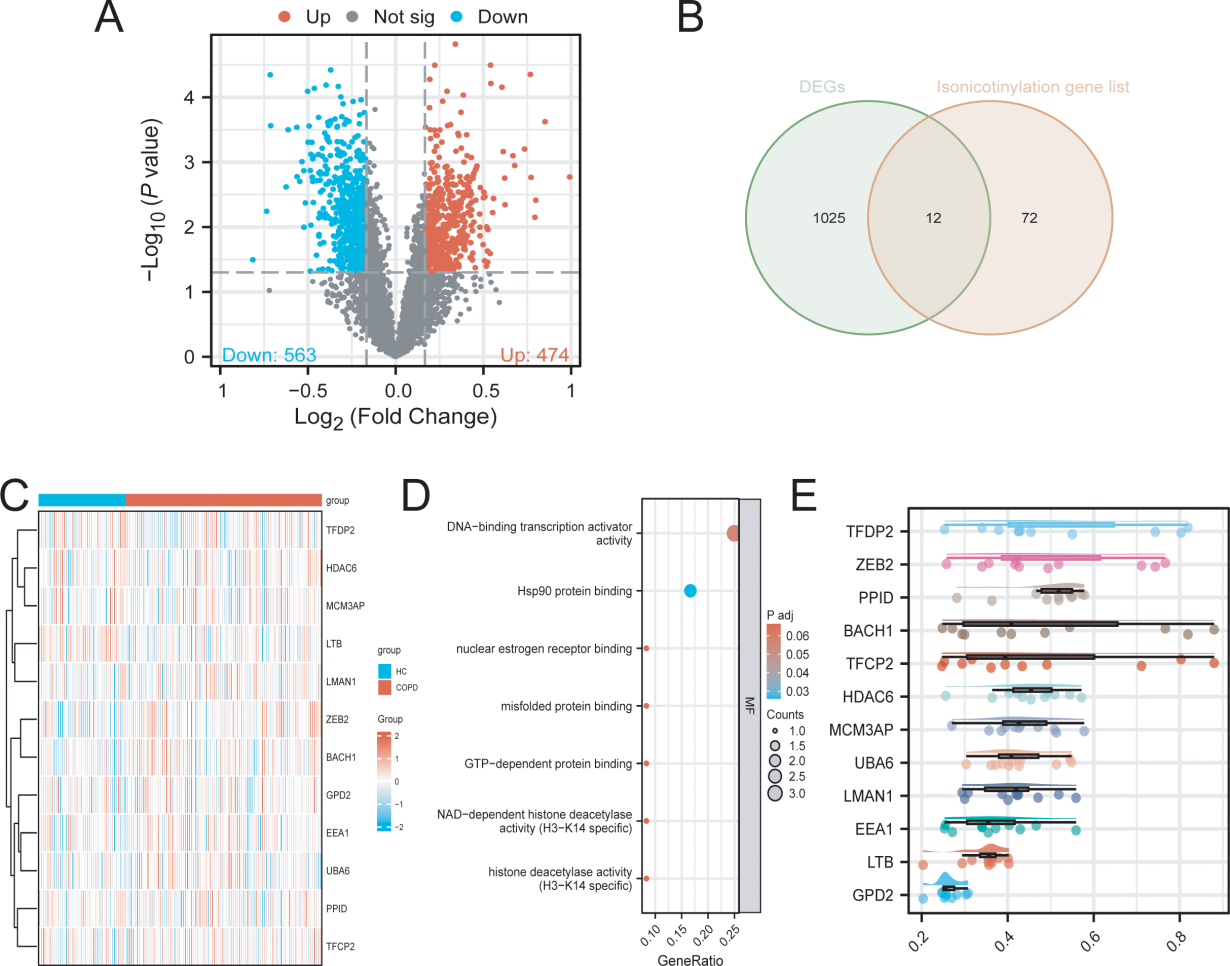
Identification of Kinic-related DEGs in COPD. **(A)** Volcano plot displaying DEGs in GSE42057. **(B)** Venn diagram illustrating the intersection between all DEGs and the Kinic-related gene set, yielding 12 core genes. **(C)** Heatmap depicting the expression patterns of the 12 Kinic-related DEGs across COPD and HC samples. **(D)** GO analysis of the 12 Kinic-related DEGs. **(E)** Functional enrichment analysis of the 12 Kinic-related DEGs.

Differential expression analysis of GSE42057 identified 1,037 DEGs between the COPD and HC groups (**Figure 1A**). Intersection of this data set with the Kinic-related gene set yielded 12 significant isonicotinylation-related DEGs (**Figure 1B**). Next, the expression patterns of these 12 DEGs in GSE42057, coupled with their molecular functions and gene feature importance, were assessed (**Figures 1C–E**).

### 3.2 Identification of neutrophil-related DEGs in COPD patients

**Figure 2:**
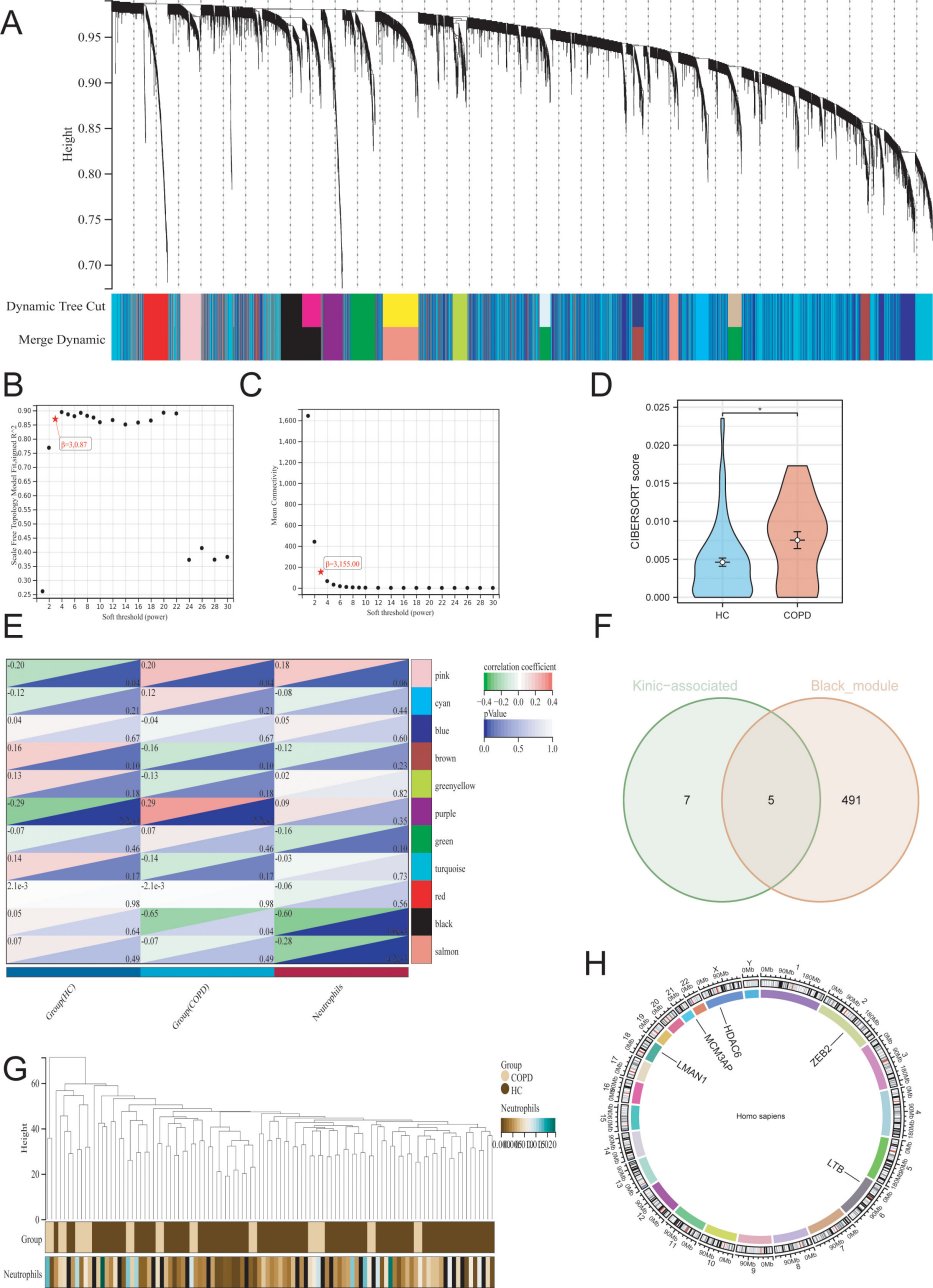
WGCNA identifies a neutrophil-correlated module and defines the KN signature. **(A)** Cluster dendrogram of genes from GSE44962, with colors indicating assigned modules. **(B, C)** Scale-free topology analysis and mean connectivity analysis for selecting the soft-thresholding power (β). **(D)** Box plot comparing the CIBERSORT-estimated neutrophil infiltration score between the COPD and HC groups in GSE44962. **(E)** Heatmap of module-trait relationships. **(F)** Venn diagram of the intersection between the genes in the neutrophil-correlated black module and the 12 Kinic-related DEGs, defining the five-gene KN-associated signature. **(G)** Tree dendrogram of neutrophil infiltration in the black module. **(H)** Chromatin localization of the five KN-associated DEGs.

CIBERSORT analysis of GSE55962 confirmed a significant increase in neutrophil infiltration in COPD samples compared to HC samples (**Figure 2D**). WGCNA constructed a co-expression network and identified a specific module (black) that showed the strongest positive correlation with the neutrophil score (**Figures 2A–C, E, G**). This module contained 496 genes functionally enriched in neutrophil activation and immune responses. After intersection with Kinic-associated DEGs, we identified five KN-associated DEGs, namely, HDAC6, ZEB2, MCM3AP, LMAN1, and LTB (**Figures 2F, H**).

### 3.3 Identification of a KN-associated diagnostic model for COPD patients

**Figure 3:**
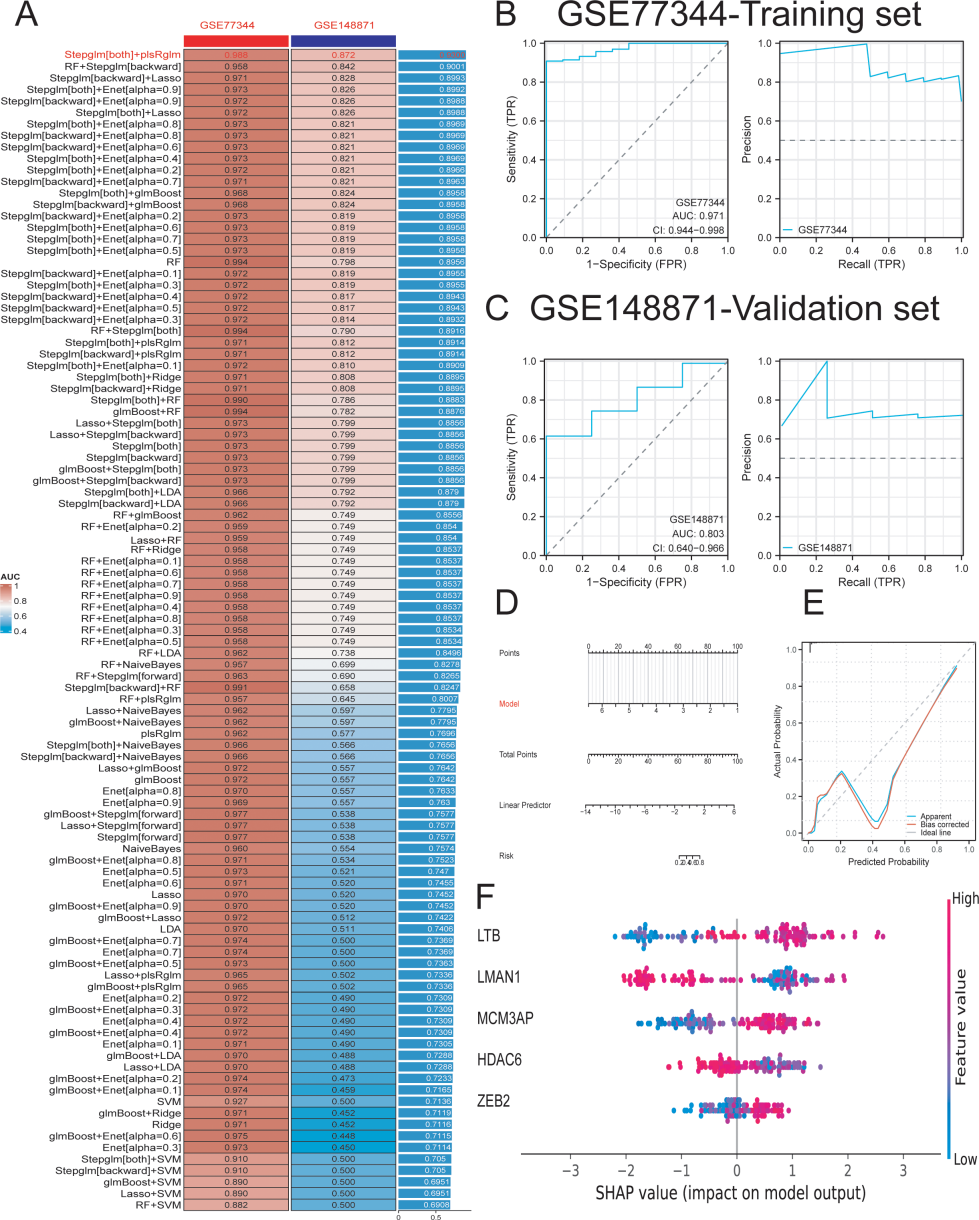
Development and validation of a KN-associated gene signature diagnostic model for COPD. **(A)** Performance metrics of 132 machine learning algorithm combinations during 10-fold cross-validation on the COPD bulk profiles. **(B, C)** ROC and PR curves of the optimal model. **(D, E)** Nomogram and calibration plot for the diagnostic model. **(F)** SHAP summary plot ranking the contribution of each KN-associated signature gene to the model output.

In the COPD training set and validation set, 132 machine learning model combinations confirmed that the Stemglm(both)+plsRglm model built on this signature achieved an AUC of 0.9300 (**Figure 3A**). This performance robustly distinguished the pathogenesis of COPD in both the training set (AUC = 0.971) and the validation set (AUC = 0.803) (**Figures 3B, C**). The calibration curve of the associated nomogram showed excellent agreement (**Figures 3D, E**). The SHAP summary plot unequivocally identified LTB as the most influential feature driving the model’s predictive power, establishing it as the central hub gene of the KN-associated axis for further analysis (**Figure 3F**).

### 3.4 Identification of KN-associated molecular subgroups in COPD patients

**Figure 4:**
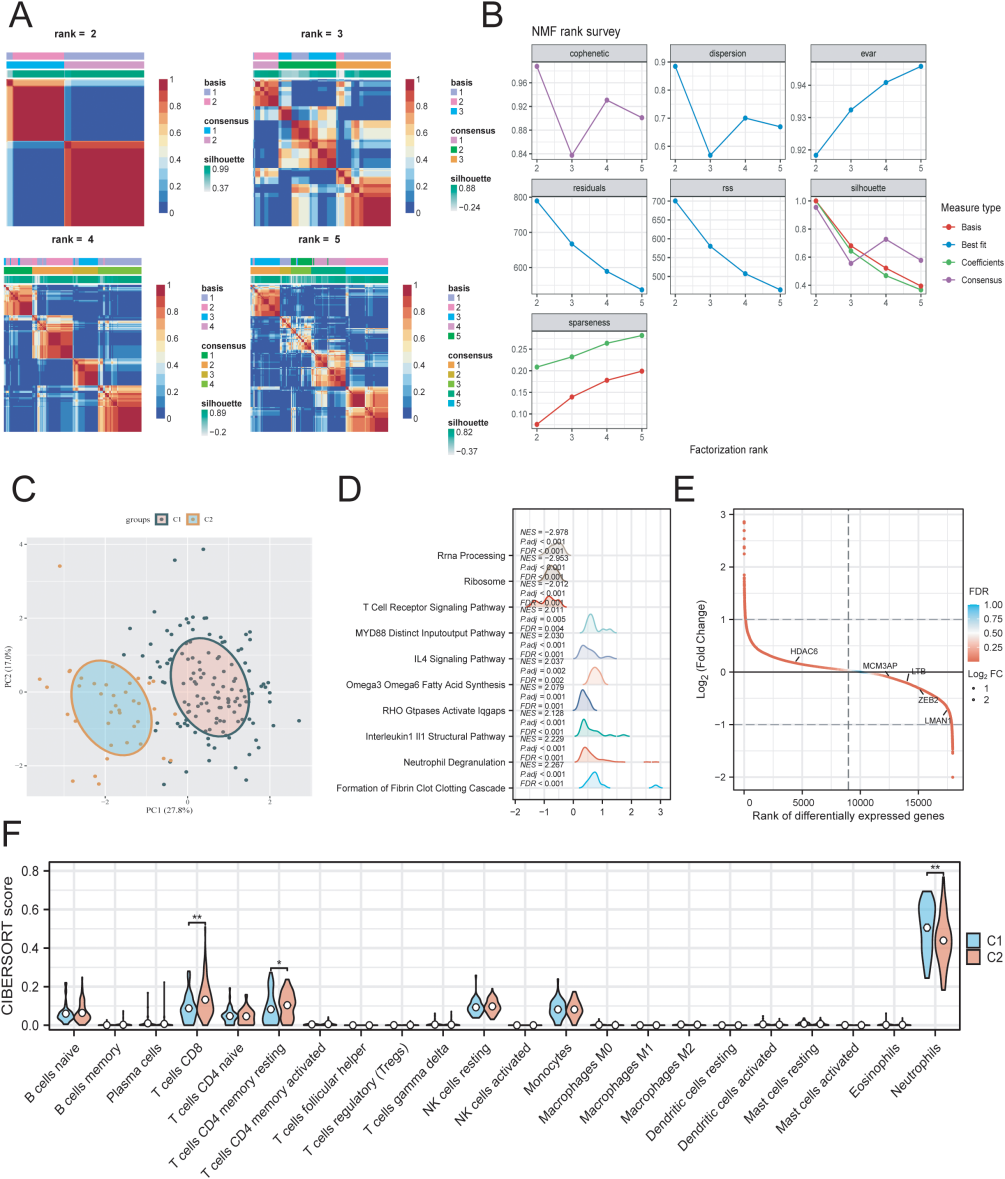
Molecular subtyping of COPD patients based on the KN-associated gene signature. **(A, B)** Consensus matrix and cophenetic correlation coefficient plot from NMF analysis supporting k=2 as the optimal number of clusters. **(C)** PCA plot showing clear separation between the two identified subtypes: C1 and C2. **(D)** GSEA plots showing representative pathways enriched in the C1 subtype compared with C2. **(E)** Heatmap comparing the expression levels of the five KN-associated gene signatures between the C1 and C2 subtypes. **(F)** CIBERSORT infiltration levels of key immune cells between the C1 and C2 subtypes.

NMF consensus clustering based on the five KN-associated gene signatures in GSE77344 robustly classified COPD patients into two molecular subtypes: C1 and C2 (**Figures 4A, B**). PCA confirmed a clear separation between the two subtypes (**Figure 4C**). All five KN-associated gene signatures were significantly downregulated in the C1 subtype compared to the C2 subtype (**Figure 4E**). GSEA revealed that the C1 subtype was markedly enriched in pathways related to neutrophil degranulation, IL-4 signaling, and epigenetic modification in C1 compared to the C2 subtype (**Figure 4D**). CIBERSORT analysis further indicated that C1 had a higher estimated infiltration of activated T cells and neutrophils (**Figure 4F**).

### 3.5 Single-cell atlas of neutrophils in COPD

**Figure 5:**
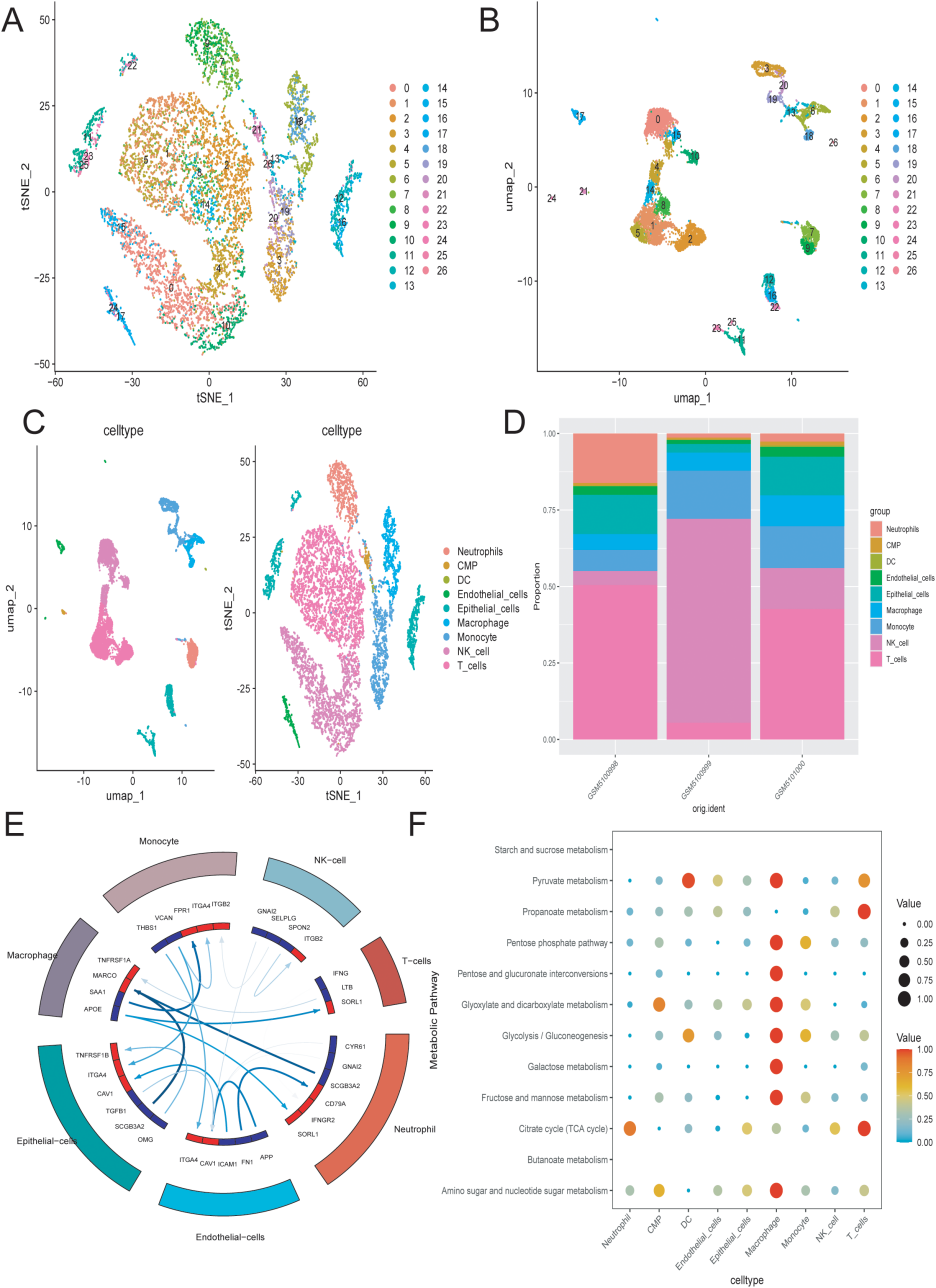
Single-cell characterization of the immune microenvironment in COPD peripheral blood. **(A, B)** UMAP and t-SNE visualizations of 26 cell clusters. **(C)** UMAP and t-SNE visualizations of 9 major cell types. **(D)** The proportion of each cell type across all samples. **(E)** Circle plot of cell-cell communication networks inferred by CellChat. **(F)** Heatmap of metabolic pathway activity scores across different cell types, analyzed using scMetabolism.

Analysis of scRNA-seq data (GSE167295) identified 26 major cell clusters, annotated as nine cell types (**Figures 5A–C**). T cells and NK cells constituted the largest proportions of the identified cell populations (**Figure 5D**). Cell-cell communication analysis suggested strong interactions between neutrophils and macrophages (**Figure 5E**). In addition, metabolic heterogeneity revealed a unique metabolic pathway in neutrophils (**Figure 5F**).

### 3.6 KN-associated signature in neutrophils in COPD patients

**Figure 6:**
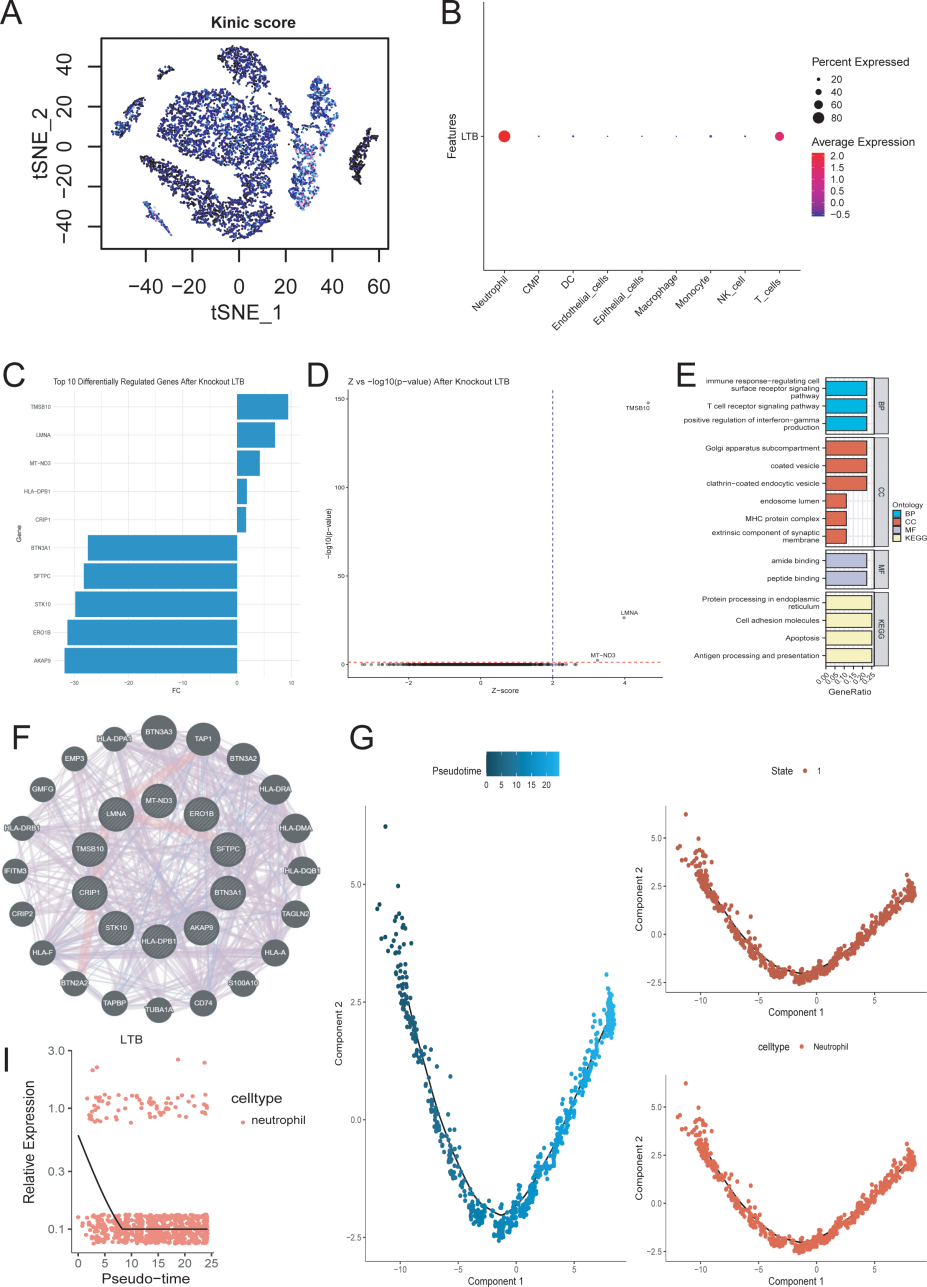
Functional characterization of the KN-associated axis in neutrophils of COPD patients. **(A)** AUCell scores for the Kinic gene set across major cell types. **(B)** Feature plot demonstrating specific expression of LTB in the neutrophil cluster. **(C–F)** Results of the *in silico* knockout of LTB in neutrophils. **(G, I)** Plots showing the expression dynamics of LTB along the neutrophil pseudotime trajectory.

AUCELL analysis revealed the activation of Kinic patterns in neutrophils (**Figure 6A**). Expression analysis at single-cell resolution confirmed that LTB expression was predominantly and specifically localized to the neutrophil cluster, with minimal expression in other cell types (**Figure 6B**). Pseudotime trajectory analysis of neutrophils revealed a differentiation continuum from a resting state to an activated state (**Figure 6G**). The expression of LTB progressively increased along the pseudotime trajectory, correlating with the acquisition of an activated phenotype (**Figure 6I**). *In silico* knockout of LTB in neutrophils perturbed a gene network significantly enriched in functions related to inflammation and epigenetic modifications (**Figures 6C–F**).

### 3.7 Drug enrichment targeting the KN-associated signature for the treatment of COPD patients

**Figure 7:**
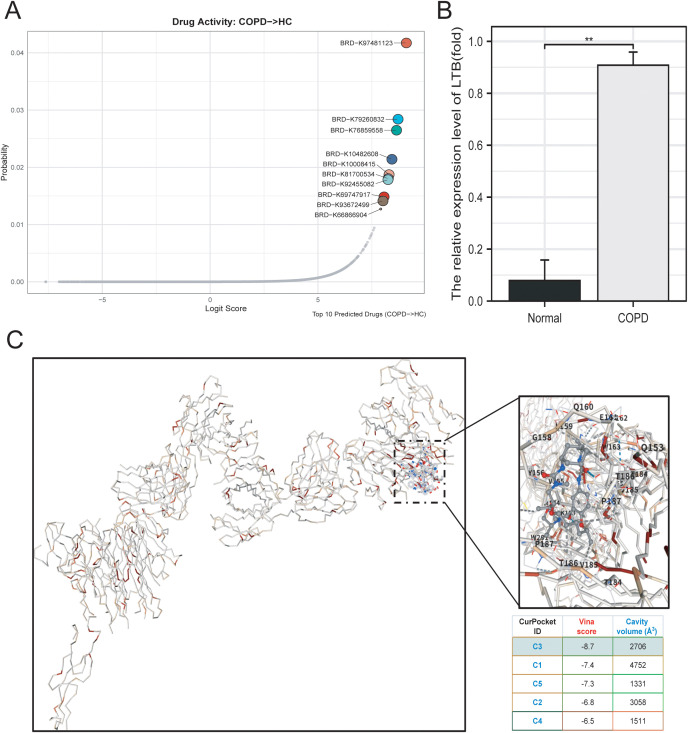
AI-driven drug screening and clinical validation. **(A)** Workflow and result of the DrugReflector screening. **(B)** q-RT-PCR) validation showing significantly elevated LTB mRNA levels in peripheral blood mononuclear cells (PBMCs) from COPD patients (n = 6) compared with healthy controls (HC, n = 6). **(C)** Molecular docking model depicting the predicted binding pose and interaction between BRD-K97481123 and the LTB protein. ** p < 0.01.

The DrugReflector screening of the COPD signature against the cMAP database identified BRD-K97481123 as the top candidate compound predicted to reverse the disease-associated gene expression profile (**Figure 7A**). Molecular docking simulations predicted stable and favorable binding interactions between BRD-K97481123 and the LTB protein, with a binding energy of -8.7 kcal/mol, suggesting high affinity and potential inhibitory activity (**Figure 7C**). In addition, *in vitro* analysis demonstrated increased expression patterns of LTB in the COPD group compared to the HC group (**Figure 7B**).

## 4 Discussion and conclusion

This integrative, AI-augmented multi-omics study potentially deciphers a novel molecular axis linking KN-associated biology in COPD. We identified five KN-associated gene signatures that could potentially guide the molecular stratification and diagnosis of COPD patients. In addition, LTB was identified as the central pathogenic factor among the five KN-associated gene signatures in COPD patients. Furthermore, we nominated a potential therapeutic compound, thereby providing a novel avenue for COPD clinical translation.

LTB is a well-established member of the tumor necrosis factor (TNF) superfamily, primarily recognized for its crucial role in the development and organization of secondary lymphoid organs (Saeki and Yokomizo, 2017). Its signaling through the LTβR receptor is pivotal in structuring immune responses (Subramanian et al., 2017). In the context of COPD, LTB has been implicated in the formation of tertiary lymphoid structures, such as inducible bronchus-associated lymphoid tissue (iBALT), which is a hallmark of advanced COPD and contributes to persistent local inflammation and disease progression (Larsson, 2008). Kinic, a recently characterized form of lysine acylation in which an isonicotinoyl moiety is transferred, has emerged as a novel metabolic sensor (Zhou et al., 2026). Previous studies have identified that LTB can undergo Kinic modification during liver cancer progression; however, the mechanisms underlying Kinic in COPD have not yet been clarified (Li et al., 2024). Moreover, there is a notable lack of evidence demonstrating significant LTB expression within neutrophils themselves in any disease context, let alone in COPD. This gap underscores a potential blind spot in the current understanding of neutrophil biology and their contribution to inflammatory networks.

In conclusion, we constructed a novel KN-associated molecular axis involved in COPD pathogenesis through an AI-driven multi-omics approach. The five-gene KN-associated signature serve as a potential diagnostic tool and reveal patient molecular and immune heterogeneity. In addition, LTB was established as a neutrophil-centric pathogenic factor in COPD. The identification of BRD-K97481123 as a potential LTB-targeting therapy offers a new translational avenue. These findings illuminate previously unrecognized KN-associated mechanisms in COPD, providing fresh insights for biomarker development and targeted therapeutic strategies. However, this study has several limitations. For instance, the direct Kinic mechanism in COPD has not yet been clarified. Future studies should focus on addressing the direct role of Kinic mechanisms in the pathogenesis of COPD using preclinical studies. In addition, although LTB was identified as a protein that can undergo Kinic modification in a previous study, the specific mechanisms of Kinic-modified LTB in neutrophils and the differential expression of LTB should be further investigated in preclinical COPD models. Furthermore, as LTB is a secreted protein, its differential expression in pulmonary tissues and peripheral blood should be evaluated in clinical trials to enhance its corresponding clinical translational potential as a liquid biopsy biomarker for COPD patients. Moreover, the diagnostic model and molecular subgroups of COPD patients identified in our study should be validated in clinical trials to assess patient clinical characteristics and improve the model’s authenticity. Finally, preclinical studies and clinical trials should be performed to evaluate the efficacy and safety of BRD-K97481123 in the treatment of COPD and to clarify its corresponding pharmacological mechanism.

## Data Availability

The datasets analyzed during the current study are publicly available in the Gene Expression Omnibus (GEO) database (https://www.ncbi.nlm.nih.gov/geo/) under the accession numbers: GSE42057, GSE44962, GSE77344, GSE148871, and GSE167295. The isonicotinylation (Kinic)-related gene signature was obtained from published literature. All code and analysis scripts used in this study are available from the corresponding author upon reasonable request.
